# Outcome and characteristics of nonsecretory multiple myeloma compared with secretory multiple myeloma: a retrospective multicenter study from China

**DOI:** 10.1186/s12885-023-11223-4

**Published:** 2023-10-02

**Authors:** Hailu Sun, Aijun Liu, Lihong Liu, Wei Wang, Zhen Cai, Hua Yan, Lijuan Chen, Guangxun Gao, Fang Wang, Aijun Liao, Bing Chen, Jia Feng, Juan Li, Dong-Ping Huang, Da Gao, Qi-Ke Zhang, Jun Luo, Rong Fu, Juan Du, Jin Lu

**Affiliations:** 1 Peking University People’s Hospital, Peking University Institute of Hematology, National Clinical Research Center for Hematologic Disease, Beijing Key Laboratory of Hematopoietic Stem Cell Transplantation, Beijing, P.R. China; 2 Department of Hematology, Beijing Chaoyang Hospital, Capital Medical University, Beijing, P.R. China; 3 Department of Hematology, The Fourth Hospital of Hebei Medical University, Shijiazhuang, Hebei P.R. China; 4 Department of Hematology, The Affiliated Hospital of Qingdao University, Qingdao, Shandong P.R. China; 5 Bone Marrow Transplantation Center, the First Affiliated Hospital, Zhejiang University School of Medicine, Hangzhou, Zhejiang P.R. China; 6 Department of Hematology, Ruijin Hospital, Shanghai Jiaotong University School of Medicine, Shanghai, P.R. China; 7 Department of Hematology, Jiangsu Province Hospital, The First Affiliated Hospital With Nanjing Medical University, Nanjing, Jiangsu P.R. China; 8 Department of Hematology, Xijing Hospital, Air Force Medical University, Xi’an, Shanxi, P.R. China; 9 Department of Hematology, The First Affiliated Hospital of Zhengzhou University, Zhengzhou, Henan P.R. China; 10 Haematology Department of Shengjing Hospital, China Medical University, Shenyang, Liaoning P.R. China; 11 Department of Hematology, The Affiliated Drum Tower Hospital of Nanjing University Medical School, Nanjing, Jiangsu P.R. China; 12 Department of Hematology, Peking University Shenzhen Hospital, Shenzhen, Guangdong P.R. China; 13 Department of Hematology, The First Affiliated Hospital of Sun Yat-Sen University, Guangzhou, Guangdong P.R. China; 14 Department of Hematology, Yijishan Hospital, The First Affiliated Hospital of Wannan Medical College, Wuhu, Anhui P.R. China; 15 Department of Hematology, the Affiliated Hospital of Inner Mongolia Medical University, Hohhot, Inner Mongolia P.R. China; 16 Department of Hematology, People’s Hospital of Gansu Province, Lanzhou, Gansu P.R. China; 17 The First Affiliated Hospital of Guangxi Medical University, Nanning, Guangxi P.R. China; 18 Department of Hematology, Tianjin Medical University General Hospital, Tianjin, P.R. China; 19 Department of Hematology, Myeloma & Lymphoma Center, Shanghai Changzheng Hospital, Naval medical University, Shanghai, P.R. China

**Keywords:** Multiple myeloma, Non secretory, Clinical feature, FLC, Prognosis feature

## Abstract

**Background:**

Nonsecretory multiple myeloma (NSMM) is a rare type of multiple myeloma (MM). Few studies have described the clinical features and outcomes of NSMM in novel agents. Additionally, the prognostic characteristics have remained controversial in recent years.

**Purpose:**

To investigate the clinical and prognostic features of NSMM and explore the prognostic value of involved free light chain (FLC) levels in NSMM patients in the Chinese population.

**Methods:**

We retrospectively enrolled 176 newly diagnosed NSMM cases between January 2005 and December 2021 from 19 clinical centers in China. The control group was selected using a 1:4 propensity score matching technique of newly diagnosed secretory MM, with age, sex and diagnosis time as the matching variables.

**Results:**

The median age of NSMM patients was 60 years, and 22.6% of patients were classified as ISS stage 3. The ORR of the NSMM patients was 87.4%, and the CR was 65.8%. Compared to the matched secretory MM patients, more NSMM patients achieved CR after first-line treatment (65.8% vs. 36%, *p* = 0.000). The ORR of first-line treatment was not significantly different between NSMM and secretory MM (89.45% vs. 84.7%, *p* = 0.196). The first-line PFS was 27.5 m and 23 m (*p* = 0.063), and the median OS was 81 m and 70 months (*p* = 0.401). However, for CR-achieved NSMM and CR-not-achieved NSMM patients, the median PFS was 37 m vs. 16 m (*p* = 0.021), while the median OS showed no difference (107 m vs. 87 m, *p* = 0.290). In multivariate analysis, the significant factors for PFS were age ≥ 65 and ISS-3. ISS-3 was the only independent prognostic factor of OS. The iFLC ≥ 50 mg/L group had a high ORR of 97.3%, and the median PFS and OS were 48 m and NR, respectively. Compared to the matched secretory MM, the iFLC ≥ 50 mg/L group also showed more CR and longer OS (NR vs. 70 m, *p* = 0.006) and PFS (48 m vs. 23 m, *p* = 0.003).

**Conclusions:**

Our results revealed that Chinese NSMM patients are younger and have a higher CR but not superior survival. The subgroup of NSMM patients with iFLC ≥ 50 mg/L had better outcomes than secretory MM.

**Supplementary Information:**

The online version contains supplementary material available at 10.1186/s12885-023-11223-4.

## Background

Multiple myeloma (MM) is a malignant plasma disease characterized by uncontrolled proliferation of monoclonal plasma cells in the bone marrow and secretion of monoclonal immunoglobulins (M protein) in serum and/or urine. 3–5% [1, 2] MM without detectable M protein by immunofixation in serum and urine is defined as nonsecretory multiple myeloma (NSMM) [1, 3]. The clinical outcome of NSMM has been assessed in some small-scale studies [4–10] and a few large-scale studies [1, 7, 11], with most patients not using novel agents. There were many paradoxical outcomes of these studies. First, the prognosis of NSMM is controversial compared with that of contemporary secretory multiple myeloma [5–11]. Second, t(11;14) was the most frequently observed cytogenetic abnormality in NSMM patients. In NSMM, t(11;14) is related to inferior survival [9]. In most studies of multiple myeloma, t(11;14) is a sign of relatively intermediate risk or good risk [12], and this discrepancy is unknown. Third, with the widespread use of novel drugs, there is a trend of better NSMM outcomes. However, the response rate did not improve. In addition, abnormal serum kappa or lambda FLC concentrations are found in NSMM patients [4]. However, the impact of sFLC on prognosis remains unclear in the era of novel agents. The prognosis of multiple myeloma is related not only to different disease types but also to different treatment modes.

Here, we enrolled 176 NSMM patients from multiple centers in China, analyzed them retrospectively and compared them with contemporary secretory multiple myeloma (secretory MM) to answer the above question in the context of novel agent therapy. The study was approved by the IRB of Peking University People’s Hospital. (2022PHB250-001).

### Patients and methods

A total of 176 newly diagnosed NSMM patients from 19 centers in China were included. These patients were diagnosed and treated between 2005 (the year bortezomib was approved in China) and 2021. All of them met the diagnostic criteria of NSMM according to IMWG2014 criteria [3]. The following clinical data were recorded as previously described: routine blood tests, creatinine, lactate dehydrogenase, serum protein electrophoresis (SPE), (serum and urine) immunofixation electrophoresis (IFE), extramedullary plasmacytoma (EMP), bone marrow plasma cell (BMPC) percentage, immunophenotype of plasma cell, bone marrow chromosome karyotyping by G-banding and FISH examination for RB1 deletion, 1q21 amplification, IgH rearrangement, P53 deletion, and D13S319 deletion [13].

We matched NSMM with secretory multiple myeloma (secretory MM) at a ratio of 1:4 as a control matched for age, sex, and year of diagnosis. A total of 554 secretory MM patients from Peking University People's Hospital were selected randomly. The clinical data of the two groups were compared carefully, including the characteristics mentioned above.

### FLC assay

Serum free light chain (sFLC) was measured using freelite reagents [The Binding Site (TBS), Birmingham, UK] before treatment. The normal value range of the FLC was κ 3.3 ~ 19.4 mg/L, λ 5.7 ~ 26.3 mg/L, and the normal range of the sFLC ratio was 0.26 ~ 1.65. The serum free light chain difference (dFLC) is defined as the difference between the involved free light chain (iFLC) and uninvolved free light chain according to the type of light chain involved.

### Definition and response assessment

NSMM patients were defined as those with no detectable abnormalities on serum or urine immunofixation, according to IMWG 2014 [3]. Patients were staged by ISS and DS. Progression free survival (PFS) was defined as the time from diagnosis to disease progression or death. Overall survival (OS) was defined as the time from diagnosis to death from any cause.

The response evaluation was performed according to IMWG2016 [14]. Complete response (CR) was defined as the disappearance of any soft tissue plasmacytomas and < 5% plasma cells in bone marrow aspirates, and normal sFLC ratio if sFLC was measurable at diagnosis. Partial response (PR) was considered a ≥ 50% decrease in the difference between involved and uninvolved FLC levels or a ≥ 50% reduction in plasma cells if serum free light assay is unmeasurable, provided baseline bone marrow plasma-cell percentage was ≥ 30%. In addition to these criteria, if present at baseline, there was a ≥ 50% reduction in the size of soft tissue plasmacytomas. Progressive Disease (PD) was considered: 1) a ≥ 25% increase in the dFLC level and the absolute value shall be increased by > 100 mg/L; 2) a ≥ 25% increase in the proportion of BMPC and the absolute value shall be increased by ≥ 10%; 3) new soft tissue plasmacytoma appears, or a ≥ 50% increase from lowest point in SPD of > 1 lesion, or a ≥ 50% increase in the long axis of the original > 1 cm lesion in short axis; 4) a ≥ 50% increase in circulating plasma cell with ≥ 200 cells per μl, if this is the only measure of disease.

Overall response rate (ORR) was considered the proportion of patients whose efficacy evaluation reached that of PR or above PR.

### Statistical analysis

The chi-square test and Fischer´s exact test were used to assess categorical variables. The PFS and OS curves were drawn by the Kaplan‒Meier method and compared by the log-rank test for univariate analysis of categorical variables. Logistic regression combined with single factor Cox analysis was performed to analyze continuous variables. To estimate the relationship between OS and the exploratory variables, Cox proportional hazard regression was used and is presented as hazard ratios (HRs) and 95% confidence intervals (CIs). All variables with a *P* value < 0.1 were included in the Cox analysis. Differences between survival curves were analyzed using the Kaplan‒Meier method and the log-rank test. All *P* values were bilateral, and a *P* value < 0.05 was considered statistically significant. All statistical analyses were performed using SPSS 26.0 (SPSS Inc., Chicago, IL, USA).

## Results

### Baseline characteristics of the NSMM

We enrolled 176 patients who met the criteria for NSMM. The median age was 60 y (range, 23–85 y), with 76.7% of patients younger than 65 y, and 54.3% were male. The percentages of patients classified as ISS stage 1, 2, and 3 were 42.3%, 32.1%, and 22.6%, respectively. Thirty-five percent of patients had anemia (Hb < 10 g/dl), and 4.3% of patients had renal impairment (creatinine > 2 mg/dl). Elevated serum LDH was found in 23% of the patients. Forty-seven out of 171 patients were found to have EMP, including paraosseous and soft-tissue plasmacytoma (1 liver, 1 spleen, 1 pleura).

The median BMPC percentage at diagnosis was 23.5%, with 27.3% patients having BMPC < 10%. For cytogenetic analysis, 17% of patients had deletion 17p, and 51.6% of patients had IGH rearrangement frequently detected in NSMM. Overall, 28/56, 5/53, and 6/52 patients had t(11;14), t(4;14), and t(14;16), respectively. Ten out of 81 patients had abnormal karyotypes, including 2 hypodiploid karyotypes, 5 pseudodiploid karyotypes, and 3 hyperdiploid karyotypes. (Details are shown in Table 1).

**Table 1:** Baseline patient characteristics of NSMM and secretory MM

	NSMM(176)	MM(554)	*p*
Gender(M)	95(54.3%)	290(52.3%)	0.654
Median age	60(23–85)	59(23–87)	0.557
Age > 65	41(23.3%)	140(25.3%)	0.597
**Hb < 100 g/L**	**55(35.0%)**	**307(58.4%)**	**0.000**
**Scr > 177umol/L**	**6(4.3%)**	**86(16.4%)**	**0.000**
LDH > 240U/L	34(22.8%)	106(21.7%)	0.768
EMP	47(27.5%)	121(25.4%)	0.597
**ISS I-II/III**	**123/36(77.4%/22.6%)**	**310/227(57.8%/42.3%)**	**0.000**
**BMPC < 10%**	**37(26.2%)**	**84(15.8%)**	**0.004**
**Del17p**	**16(17.0%)**	**39(8.7%)**	**0.015**
IGH rearrangement	48(51.6%)	273(60.1%)	0.128
**T(11;14)**	**28(50.0%)**	**82(21.9%)**	**0.000**
T(4;14)	5(7.9%)	59(15.7%)	0.231
**T(14;16)**	**6(11.5%)**	**3(0.8%)**	**0.000**
Gain of 1q21	35(38.5%)	201(44.9%)	0.262
Del(13q14)	25(38.5%)	173(39.9%)	0.830
Del(13q14.4)	18(30.5%)	177(39.4%)	0.186
**Abnormal Karyotype**	**10(12.3%)**	**131(30.0%)**	**0.001**
Treatment			0.375
PI based	95(56.2%)	265(49.7%)	
IMiDs based	27(16.0%)	85(15.9%)	
PI + IMiDs	37(21.9%)	135(25.3%)	
Others	10(5.9%)	48(9.0%)	
**SCT**	**26(18.4%)**	**116 (27.8%)**	**0.028**
Response			0.000
ORR	102(89.4%)	360(84.7%)	
**CR**	**75 (65.8%)**	**153 (36.0%)**	**0.000**

*EMP* extramedullary plasmacytoma, *BMPC* bone marrow plasma cell, *PI* Proteasome inhibitors, *IMiDs* Immunomodulatory drugs, *SCT* stem cell transplantation

When compared to 554 matched secretory MM patients, there was no difference in age or sex. NSMM was more often staged in ISS-I (45.3% vs. 23.3%) than in ISS-II (32.1% vs. 34.5%) or ISS-III (22.6% vs. 42.3%). The NSMM group had less anemia (35.0% vs. 58.4%, *p* = 0.000) and less renal dysfunction (4.5% vs. 16.4%, *p* = 0.000). There were more patients with BMPC less than 10% among NSMM patients (27.3% vs. 15.8%, *p* = 0.004). A high incidence of t(11;14) was observed in NSMM patients (50% vs. 21.9%, *p* = 0.000). We also detected a higher percentage of deletion 17p in NSMM patients (17.0% vs. 8.7%, *p* = 0.015).

More than 90% of patients received induction treatment containing PI or IMiDs as first-line treatment. A total of 56.2% accepted therapies based on PI, including VCd (27.3%), Vd (8.4%), and VAd (13.7%). A total of 16.0% received treatment based on IMiDs. A total of 21.9% were treated with PI + IMiDs. Only 5.9% received M2, MP, etc. Furthermore, 18.4% received autologous stem cell transplantation (ASCT) after induction therapy.

**Fig. 1:**
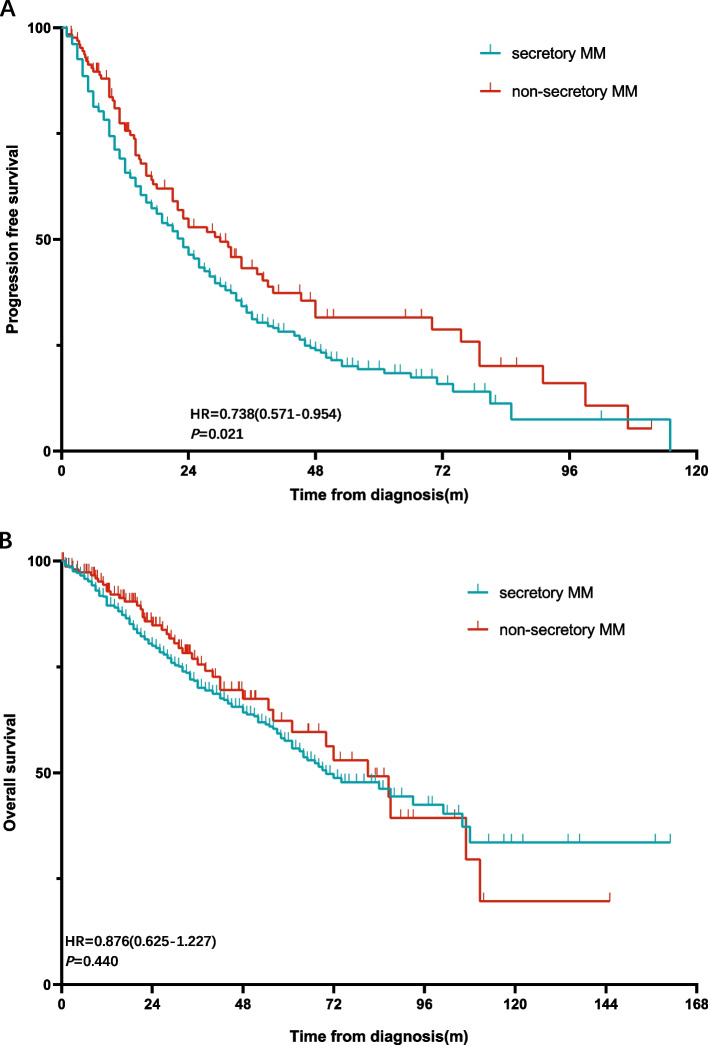
**A** the K-M curve of PFS analysis in NSMM and secretory MM. **B** the K-M curve of OS analysis in NSMM and secretory MM

The first-line PFS was 27.5 m and 23 m (*p* = 0.063). The median OS for NSMM patients was 81 months, compared to 70 months for matched secretory MM patients (*p* = 0.401) (Fig. 1). The estimated 5-year survival was 56.2% vs. 49.3% (*p* = 0.170). For patients received ASCT, the estimated 3-years PFS was 49.6% vs. 57.0%, *p* = 0.434, and the estimated 5-year OS was 76.6% vs. 75.3%, *p* = 0.842.

And there were 36 and 227 patients ranked as ISS-III in the NSMM and secretory MM group. The first-line PFS and OS showed no difference, as the median PFS was 15 m and 18 m (*p* = 0.628), the median OS was 32 m and 58 m (*p* = 0.668). But in patients ranked as ISS-I/II, the NSMM showed better PFS (37 m and 26 m, *p* = 0.023) than MM. The same trend was found in patients beyond 65 years old. NSMM patients ≤ 65y showed longer PFS(34 m and 23 m, *p* = 0.010) but not OS (87 m and 87 m, *p* = 0.135) than MM.

### Serum free light chain assay has prognostic significance

Ninety-two patients received sFLC assay at diagnosis, with 52 with elevated κ chain, 11 with elevated λ chain, and 29 with normal FLC. We separated these patients according to iFLC level, as iFLC ≥ 50 mg/L and iFLC < 50 mg/L. There were 50 and 42 cases, respectively. The iFLC ≥ 50 mg/L group had more patients younger than 65 years and less elevated LDH (Details in Table 2). The induction treatment mode and the use of autologous stem cell transplantation (ASCT) showed no significant difference between the two subgroups. The iFLC ≥ 50 mg/L group had a higher ORR (97.3% vs. 81.1%, *p* = 0.025) and longer PFS (48 m vs. 21 m, *p* = 0.032) and OS (NR vs. 56 m, *p* = 0.007).

**Table 2:** Clinical characteristics of NSMM either with iFLC ≥ 50 mg/L

	iFLC ≥ 50 mg/L(*n* = 50)	iFLC < 50 mg/L(*n* = 42)	*P*
Range(m)	51.3–1945.0(232.5)	0.8–32.0(7.94)	
Gender(M)	29(58.0%)	23(54.8%)	0.755
Age > 65	7(14.0%)	14(33.3%)	0.028
Hb < 100 g/L	14(28.6%)	16(38.1%)	0.335
Scr > 177umol/L	2(4.0%)	1(2.4%)	
LDH > 240U/L	3(6.1%)	12(28.2%)	0.004
BMPC < 10%	13(26.0%)	9(21.4%)	0.609
Del17p	9(29.0%)	2(8.7%)	
T(11;14)	16(53.3%)	8(44.4%)	0.551
Gain of 1q21	12(32.4%)	14(51.9%)	0.118
Response			
ORR	36(97.3%)	30(81.1%)	0.025
CR	26(70.3%)	20(54.1%)	0.150

For patients treated with immunomodulatory drugs (IMiDs) as first-line therapy, the prognostic difference between the iFLC ≥ 50 mg/L and iFLC < 50 mg/L groups could be eliminated. However, for patients treated with proteasome inhibitors (PIs) only, the prognosis difference between the two subgroups remained. For patients treated with IMiDs, the median OS was NR vs. 87 m (*p* = 0.221) in the iFLC ≥ 50 mg/L and iFLC < 50 mg/L groups. For patients treated without IMiDs, the median OS was not reached and was 56 m (*p *= 0.015) in the two groups.

**Fig. 2:**
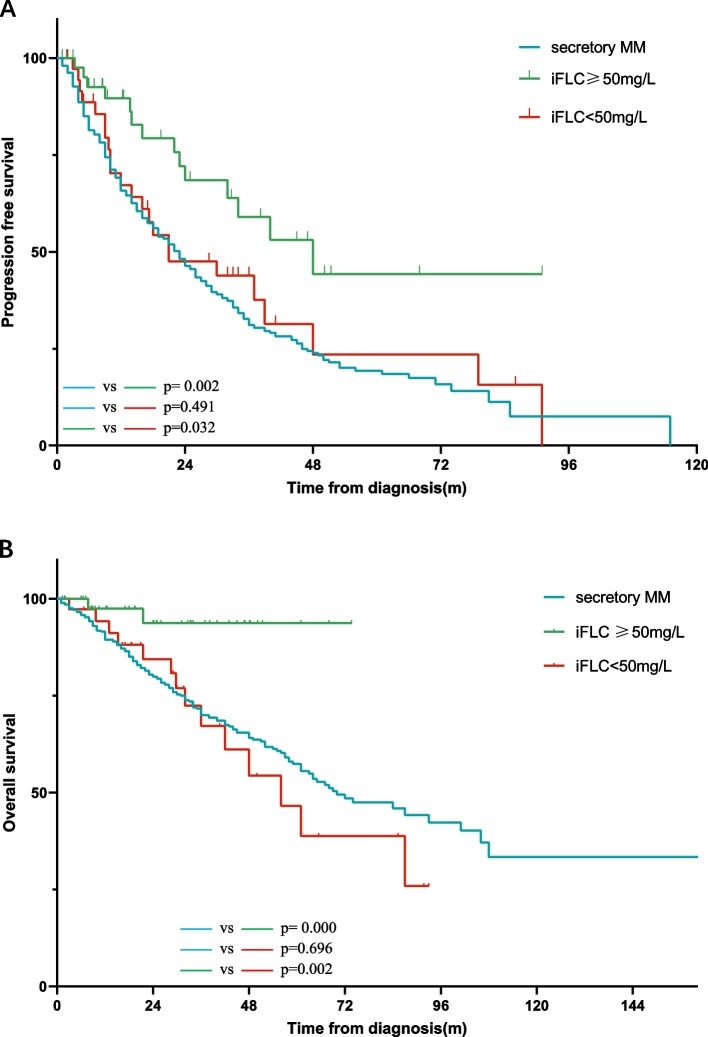
**A** the K-M curve of PFS analysis of NSMM with different iFLC levels. **B** the K-M curve of OS analysis in NSMM with different iFLC levels

Compared to the matched secretory MM group, the iFLC ≥ 50 mg/L group also showed less anemia, less ISS-III, less 1q21 amplification and more t(11;14). The iFLC ≥ 50 mg/L group had more CR after induction treatment (70.3% vs. 36.0%, *p* = 0.000). Both OS (NR vs. 70 m, *p* = 0.006) and PFS (48 m vs. 23 m, *p* = 0.003) were longer in the iFLC ≥ 50 mg/L group (Fig. 2).

### Higher CR Did Not Translate to OS Benefit in NSMM Patients

The ORR of the NSMM patients was 87.4%, and the CR was 65.8%. Among the patients with different induction therapies, there was no significant difference in the remission rate (Details showed in Table 3). More NSMM patients achieved CR after first-line treatment than secretory MM patients (65.8% vs. 36%, *p* = 0.000). The ORR of first-line treatment was not significantly different for NSMM and secretory MM (89.45% vs. 84.7%, *p* = 0.196).

**Table 3:** The remission rate according to different induction treatment

	PI (*n* = 66)	IMiDs (*n* = 15)	PI + IMiDs (*n* = 29)
ORR%	89.3	86.7	93.1
CR%	68.1	73.3	62.1
NR%	10.7	13.3	6.9

Data doesn’t include 4 cases using other treatment

After a median follow-up of 43 months, the median PFS was 27.5 months, and the median OS was 81 months. For patients receiving ASCT, the median PFS was NR compared to 24 m for patients who did not receive ASCT (*p* = 0.133). The median OS was NR for patients receiving ASCT and 81 m for the rest, *p* = 0.078.

We analyzed the prognostic effect of sex, age, diagnosis year, LDH, ISS stage, creatine, extramedullary plasmacytoma, anemia, P53 deletion, 1q21 amplification, and t(11;14) translocation using univariate and multivariate analyses. The results showed that in univariate analysis, the significant factors for OS were age ≥ 65 years, ISS-3, initial hemoglobin level < 100 g/L, LDH > 240 U/L, P53 deletion and 1q21 amplification. PFS was related to older age, ISS-3, initial hemoglobin level < 100 g/L, and 1q21 amplification. With multivariate analysis, the significant factors for PFS were age ≥ 65 and ISS-3. ISS-3 is the only independent prognostic factor of OS.

When divided depending on whether CR was achieved, the PFS of the two groups showed a significant difference in NSMM, as the median PFS was 37 m and 16 m (*p* = 0.021) for the CR-reaching NSMM group and the CR-not-reaching NSMM group, respectively, and the OS of the two groups showed no significant difference (107 m vs. 87 m, *p* = 0.290).

For NSMM patients with iFLC ≥ 50 mg/L, the median PFS showed no difference (NR vs. 48 m, *p* = 0.236) for the CR-reached NSMM group and the CR-not-reached NSMM group. The median OS was NR (*p* = 0.021) for both groups, as more than 70% of patients remained alive at the last follow-up visit.

For NSMM with iFLC < 50 mg/L, both PFS (37 m vs. 10 m, *p* = 0.284) and OS (61 m vs. 87 m, *p* = 0.959) showed no significant differences between the CR-reached and the CR-not-reached groups.

For NSMM patients who achieved CR, the median PFS showed no difference (NR vs. 37 m, *p* = 0.138) in the iFLC ≥ 50 mg/L and iFLC < 50 mg/L groups. The median OS was NR vs. 61 m, *p* = 0.019 for both groups.

For contemporary secretory MM patients, significant differences were found in both PFS (50 m vs. 15 m, *p* = 0.000) and OS (NR vs. 61 m, *p* = 0.000) between the CR-reached and CR-not-reached groups.

### Subgroup analysis

#### Extramedullary plasmacytoma

In a subgroup analysis of patients with EMP (*n* = 47) or without EMP (*n* = 124), more patients in the NSMM with EMP group were ranked as ISS-I (68.9% vs. 33.6%, *p* = 0.000), had more patients with BMPC < 10% (42.6% vs. 21.0%, *p* = 0.004), and had less anemia (14.6% vs. 43.4%, *p* = 0.001). For first-line treatment, although more patients with EMP received PI (87.2% vs. 72.6%, *p* = 0.045) and ASCT (33.3% vs. 12.9%, *p* = 0.005), the ORR and survival were not significantly different between patients with or without EMP.

#### Percentage of bone marrow plasma cells

When divided into BMPC ≥ 10% and BMPC < 10% groups, 128 patients (72.7%) had bone marrow plasma cells (BMPCs) ≥ 10%, and 48 patients (27.3%) had 0–10% BMPCs. Patients with BMPC ≥ 10% had less EMP (21.6% vs. 43.5%, *p* = 0.004) and more anemia (44.6% vs. 5.7%, *p* = 0.000), ISS-III (30.4% vs. 2.3%, *p* = 0.000), and t(11;14) translocation (56.0% vs. 0, *p* = 0.031). However, there was no significant difference in the BMPC ≥ 10% and BMPC < 10% groups for OS (81 m vs. 110 m, *p* = 0.202) and PFS (24 m vs. 32 m *p* = 0.769). In patients with BMPC ≥ 10%, there remained a significant difference in the median OS of iFLC ≥ 50 mg/L and iFLC < 50 mg/L (NR vs. 61 m, *p* = 0.038).

#### t(11;14) Translocation

T(11;14) was found to be positive in 50% of patients who underwent FISH for IgH rearrangement before treatment. All patients with t(11;14) had BMPC > 10%, while 25% of NSMM patients without t(11;14) had BMPC < 10%. Patients with t(11;14) had lower LDH levels, as all patients in this group had normal LDH levels. However, there was no significant difference in ORR between them. The median PFS was 34 m vs. 48 m for NSMM with/without t(11;14) (*p* = 0.939). However, OS for both groups was not reached (*p* = 0.448). Patients with t(11;14) showed no difference in median OS and PFS for different iFLC levels (*p* = 0.362).

#### ISS stage

Thirty-six patients were ranked as ISS III, and 123 patients were ranked as ISS I/II. There was no difference in the induction treatment mode and ASCT rate in different ISS ranks. Patients ranked as ISS-III had more EMP, renal insufficiency, anemia, BMPC > 10% and abnormal karyotypes. The median OS and PFS were inferior in patients ranked as ISS-III. The median OS was 32 m vs. 87 m, *p* = 0.004, and the median PFS was 17 m vs. 38 m, *p* = 0.007 in patients categorized as ISS-III and ISS-I/II. However, for patients ranked as ISS-III, the median OS showed no difference (NR vs. 36 m, *p* = 0.370) in the iFLC ≥ 50 mg/L and iFLC < 50 mg/L groups. The prognostic impact of iFLC ≥ 50 mg/L still existed in patients ranked as ISS-I/II, as the median OS was NR vs. 61 m (*p* = 0.021) in the iFLC ≥ 50 mg/L and iFLC < 50 mg/L groups.

#### Different diagnosis years

Focusing on the diagnosis year, NSMM patients were divided as diagnosed before 2017 and in 2017–2020 (bortezomib has been covered by insurance since 2017 in China). No difference in clinical characteristics was found between the two groups. The usage of therapy containing PI increased from 59.8% before 2017 to 94.5% in 2020, while therapy including IMiDs was nearly 40% in both groups. There was also a trend toward broader use of ASCT from 10% to 22.2% over time. Even though there was no significant change in the disease remission rate and CR rate, the prognosis of the disease has been greatly improved. The 3-year survival rates were 76.3% and 66.4% in 2017–2020 and before 2017, respectively.

## Discussion

NSMM is considered a rare type of MM, and it was excluded in most clinical trials. Therefore, the prognosis of NSMM is not well understood. Moreover, M protein cannot be found by immunofixation electrophoresis or protein electrophoresis in either serum or urine, which makes disease evaluation difficult. However, the morbidity, type and disease outcome of MM vary among different races [2]. Our study is the largest multicenter retrospective study for NSMM. For the first time, the cytogenetic characteristics were described in detail. Moreover, we first reported the prognostic value of the iFLC level.

In this retrospective study, we analyzed 176 newly diagnosed NSMM patients, and the median follow-up was 43 months. The largest sample-size study previously reported was the retrospective study of Wålinder et al*.* [1] in Sweden published in 2019. Compared to that study, the median age was 60 y for Chinese patients, with 23.3% patients older than 65 y, while the median age was 69 y and 64% patients over 65 y for that study. Anemia occurred in 35% of Chinese patients and 21% in that study. Regarding treatment, 94.1% of Chinese patients received novel agents as first-line treatment, while 54% of patients received novel agents as first-line treatment in the study of Wålinder et al*.* [1] The SCT rate was 18% vs. 43% in the two studies. The Chinese population had a high CR of 68.1%, while the CR of the Swedish population was 26%. This might be because of the wide usage of novel agents, as well as the younger median age in our study.

In our study, 92 patients received an sFLC assay at diagnosis, with 50 samples having iFLC ≥ 50 mg/L and 42 samples having iFLC < 50 mg/L. Our study showed a better prognosis of NSMM patients with iFLC ≥ 50 mg/L, either compared to NSMM with iFLC < 50 mg/L or compared to secretory MM. In the study of Wålinder et al*.* [1], the OS of NSMM with normal FLC had relatively inferior OS than NSMM with iFLC < 50 mg/L or iFLC ≥ 50 mg/L. The study indicated that the prognosis worsened as the iFLC decreased, which was consistent with our results. The younger age, the lower LDH level, and the less high-risk cytogenetic characteristics of tumor cells are the possible reasons of the favorable prognosis in the NSMM patients with iFLC ≥ 50 mg/L. However, in the study of Chawla et al*.* [7] The patients with normal FLC had superior OS than patients with abnormal FLC. This was opposite to our result. This might be because the study chose the FLC ratio as the variable, while we used the iFLC level.

For the patients receiving IMiDs as first-line induction treatment, the median OS of NSMM with different iFLC levels showed no difference. We might assume that patients with iFLC < 50 mg/L benefit more from IMiDs, which needs to be further confirmed.

Compared to secretory MM, the prognosis of NSMM showed no difference in our study. And in the study of Kumar et al*.* [11], the median overall survival was similar in NSMM and secretory MM patients after receiving SCT. This result was consistent with ours. However, in the study of Chawla et al*.* [7] the NSMM patients diagnosed between 2001 and 2012 had longer OS than contemporary secretory MM patients. The different conclusions might be because our patients were mostly diagnosed after 2012. In the study of Nandakumar et al. [9], the overall survival was relatively inferior for NSMM than for secretory MM, as NSMM was defined as negative IFE, negative SPE and iFLC < 50 mg/L.

CR is a vital prognostic factor and a crucial point for treatment [15] in MM. However, in our study, the higher CR did not translate into better survival. In the study of Chawla et al*.* [1], there was a trend toward better survival in patients achieving CR, while the median survival showed no difference. The same trend was found in the study of Wålinder et al*.* [1] In univariate analysis, survival was superior for CR, while in multiple analysis, CR was not an independent factor for survival. Because the current standard of CR in NSMM is easy to achieve, CR showed no prognostic effect in some cases. The results indicated that we should use other response assessment criteria for these cases, such as the combination of MRD and iFLC.

We also analyzed the prognosis of different subgroups of NSMM, such as t(11;14). Half of the patients had t(11;14). The occurrence was similar to that in existing studies [9, 16]. However, no significant difference was found in the median OS and PFS for t(11;14)-positive and t(11;14)-negative NSMMs. While Nandakumar et al. [9] obtained completely opposite results. The difference in sample size and ethnicity might be the reason.

For many other subgroups, a prognostic effect was not found. In the multivariate analysis, only two factors had prognostic effects. Age ≥ 65 was an independent prognostic factor for PFS, and ISS-3 was an independent prognostic factor for PFS and OS.

In conclusion, this study is the largest retrospective study of NSMM patients taking novel agents. This finding revealed that Chinese NSMM patients are younger and have higher CR but not superior survival. A subgroup of NSMM patients with iFLC ≥ 50 mg/L had better outcomes than secretory MM patients.

### Supplementary Information

**Additional file 1.**

## Data Availability

The datasets used and/or analysed during the current study are available from the corresponding author on reasonable request.

## Abbreviations

ASCT: Autologous stem cell transplantation
BMPC: Bone marrow plasma cell
CI: Confidence intervals
CR: Complete response
EMP: Extramedullary plasmacytoma
HR: Hazard ratios
IFE: Immunofixation electrophoresis
iFLC: Involved free light chain
IMiDs: Immunomodulatory drugs
MM: Multiple myeloma
M protein: Monoclonal immunoglobulins
NR: Not reached
NSMM: Noncsecretory multiple myeloma
ORR: Overall response rate
OS: Overall survival
PFS: Progression free survival
PI: Proteasome inhibitors
PR: Partial response
secretory MM: Secretory multiple myeloma
sFLC: Serum free light chain
SPE: Electrophoresis
